# Associations of Whey Protein and Lipophilic Vitamin Profiles with Texture and Colour Parameters of Organic Plain Yoghurts

**DOI:** 10.3390/foods15122087

**Published:** 2026-06-09

**Authors:** Aneta Brodziak, Jolanta Król, Paulius Matusevičius

**Affiliations:** 1 Department of Quality Assessment and Processing of Animal Products, Faculty of Animal Sciences and Bioeconomy, University of Life Sciences in Lublin, Akademicka 13, 20-950 Lublin, Poland; aneta.brodziak@up.edu.pl; 2 Department of Animal Nutrition, Faculty of Animal Sciences, Lithuanian University of Health Sciences, A. Mickeviciaus g. 9, LT-44307 Kaunas, Lithuania; paulius.matusevicius@lsmu.lt

**Keywords:** natural yoghurt, whey proteins, fat-soluble vitamins, β-carotene, texture profile analysis, CIE Lab* colour, RP-HPLC

## Abstract

In view of the importance of texture and colour characteristics for consumer acceptance of fermented dairy products, this study aimed to evaluate the associations between selected whey proteins, fat-soluble vitamins, and instrumental quality traits of organic plain yoghurts. Physicochemical analyses included acidity, total protein, fat, whey proteins (β-lactoglobulin, α-lactalbumin, bovine serum albumin, lactoferrin, and lysozyme), and fat-soluble compounds (vitamins A, D_3_, and E, and β-carotene). Selected instrumental texture and colour attributes were also determined. Whey protein fractions were associated mainly with texture characteristics, whereas lipophilic vitamins and β-carotene were more closely related to colour attributes. Total protein content was positively associated with firmness (r = 0.510, *p* ≤ 0.05), while β-lactoglobulin was related to apparent viscosity (r = 0.705, *p* ≤ 0.05). In contrast, vitamin A, vitamin D_3_, β-carotene, and fat content were negatively associated with lightness and whiteness, but positively related to yellowness and chroma. Lactoferrin and lysozyme showed stronger relationships with selected secondary texture attributes, particularly gumminess and cohesiveness. Multiple regression analysis indicated that firmness and apparent viscosity were only moderately explained by the selected compositional predictors, whereas colour attributes were more strongly associated with fat-fraction components. The remaining variability likely reflected additional microstructural and processing-related effects. Although the results should be interpreted as associations rather than direct causal relationships, the findings support the concept of protein-driven texture and fat-driven colour development in plain yoghurts and improve understanding of the relationships between bioactive milk components and instrumental plain yoghurt quality.

## 1. Introduction

### 1.1. Organic Yoghurt Production and Quality Characteristics

Milk and dairy products have become an integral component of the daily human diet due to their rich nutritional value and beneficial effects on human health. Among dairy products, fermented milk beverages have attracted particular attention because of their nutritional, functional, and probiotic properties. Yoghurt is currently one of the most widely consumed fermented dairy products worldwide and remains an important part of the human diet [1,2,3,4]. Increasing consumer interest in natural and organic foods, as well as products associated with health-promoting effects, has contributed to the growing importance of organic yoghurts on the dairy market [5,6,7]. In recent years, organic dairy products, particularly yoghurts and cheeses, have gained significant popularity worldwide, especially in European countries. The dynamic growth of the organic food sector, combined with increasing consumer demand for minimally processed and functional dairy products, creates opportunities for both industrial dairies and small-scale on-farm processing enterprises [8,9]. Nevertheless, despite increasing demand, organic yoghurts still represent a relatively niche segment of the dairy industry [10]. Moreover, precise statistical data concerning the annual volume of organic milk used specifically for yoghurt production are not publicly available, since most dairy statistics aggregate organic and conventional products within broader dairy categories. The smaller scale and fragmented nature of organic yoghurt processing additionally limit data availability.

Organic dairy products are manufactured from raw materials obtained from animals raised on certified farms under strictly regulated production systems. These systems are based on outdoor access, feeding with locally produced natural feedstuffs, and restrictions on the use of growth stimulants, hormones, synthetic vitamins, and genetically modified organisms (GMOs). Furthermore, only authorized substances and additives approved for organic food processing may be used during production [11]. These characteristics are closely associated with the composition of milk, since its individual components influence not only nutritional and health-promoting properties, but also technological suitability and the quality characteristics of dairy products [12,13].

### 1.2. Whey Proteins, Lipophilic Vitamins and Their Role in Yoghurt Quality

Contemporary studies increasingly highlight the multidirectional beneficial effects of bioactive components present in the protein and fat fractions of milk on both neonatal development and human health. These findings are progressively being utilized by the dairy industry in the development of functional dairy products. Particular attention has been given to whey proteins, which constitute approximately 20–25% of the total protein content of cow’s milk. Whey proteins exhibit the anti-inflammatory, antitumour, immunostimulatory, hypotensive, gut homeostasis, antiobesity, antidiabetic, muscle biosynthesis, osteoprotective, and radioprotective functions. Among them, lactoferrin, lysozyme, lactoperoxidase, and immunoglobulins are recognized for their antibacterial, antiviral, antifungal, and antiparasitic activities [14,15,16]. Lipophilic (fat-soluble) vitamins constitute an important group of organic compounds with high biological activity that are essential for growth and normal physiological functioning. They participate in numerous key life processes, support metabolic pathways, and enhance the activity of enzymes and catalytic proteins. Although the human body requires only small amounts of vitamins, their deficiency can have significant adverse effects on health [14,17,18,19].

Alongside nutritional and health-promoting properties, organoleptic characteristics are major determinants of consumer acceptance of fermented dairy products. In yoghurt, texture and colour are among the most important quality attributes influencing consumer preference and product perception. The quality of yoghurt is determined not only by its nutritional composition, but also by instrumental texture and colour parameters associated with gel structure, water-binding capacity, emulsion stability, and light-scattering properties [20,21].

Milk is a complex natural oil-in-water emulsion whose physicochemical and sensory properties depend on interactions between milk proteins, fat globules, and the aqueous phase. Modifications in emulsion structure and processing conditions may affect gel formation, texture, stability, and colour characteristics of fermented dairy products. Processing technologies such as homogenization, heat treatment, high-pressure processing, ultrasonication, or microfluidization may alter protein-fat interactions and emulsion microstructure, thereby influencing the final quality of yoghurt and other gelled dairy products [22,23]. Consequently, understanding the relationships between bioactive milk constituents and instrumental quality parameters is becoming increasingly important for the development of high-quality and multifunctional dairy products meeting current consumer expectations. However, despite numerous studies concerning yoghurt quality, limited knowledge is available regarding the contribution of individual whey proteins and lipophilic vitamins to the instrumental texture and colour characteristics of organic plain yoghurts. Therefore, the present study aimed to investigate the associations between these bioactive milk constituents and selected instrumental quality parameters of organic plain yoghurts. It was hypothesized that individual whey proteins would be more strongly associated with texture-related parameters, whereas lipophilic vitamins and β-carotene would show stronger associations with colour attributes of organic plain yoghurts.

## 2. Materials and Methods

### 2.1. Research Material

The research materials were 318 organic natural, plain yoghurts produced from raw milk of Simmental cows. The analysed yoghurts represented independent production batches manufactured under standardized laboratory conditions. Each batch was produced from a separate bulk milk collection obtained from certified organic dairy farms. Milk was collected from 17 farms maintaining between 8 and 25 cows. Milk collections originated from different production days, allowing inclusion of natural biological and technological variability characteristic for organic milk production. The experimental unit was the independent yoghurt production batch. Analytical replicates performed for physicochemical, texture, and colour analyses were averaged before statistical analysis. The organic origin of milk was verified based on certification of the supplying farms in accordance with European Union regulations for organic production [11]. The somatic cell count (using Somacount 150, Bentley Instruments, Chaska, MN, USA) and total bacterial count (according to Polish Standard [24]) in the raw bulk milk were determined in order to verify whether it met the requirements for hygienic and microbiological quality, i.e., SCC < 400,000 in 1 mL and TBC < 100,000 in 1 mL. The yoghurts were manufactured in laboratory conditions by the water bath method (the thermostatic method). Raw milk was heat treated at 85 °C for 30 min in a water bath. Next it was cooled to 43 °C and inoculated with the starter strains of yoghurt bacteria from Chr. Hansen (Denmark) in the amount of 0.15 g/L. Thermophilic yoghurt FD-DVS YC-380 Yo-Flex mixed strain culture containing *Streptococcus thermophilus* and *Lactobacillus delbrueckii* subsp. *bulgaricus* (YC-380) was used. The milk was incubated at 43 °C until pH = 4.6 was attained (approx. 5 h). This end-point pH corresponded closely to the isoelectric point of casein, at which the milk system transforms into a fragile acid gel [25]. Yoghurt fermentation involves a transition from a Newtonian liquid to a non-Newtonian thixotropic gel, and cooling is applied once the target pH is reached to limit further acidification and stabilize the gel structure. The final rheological properties of yoghurt depend not only on acidification itself, but also on the interactions among caseins, whey proteins, water, and fat globules within the developing matrix [26]. Then the products were cooled to 20 °C to discontinue incubation. The yoghurts were stored at 4–6 °C until the next day (about 14 h) for analysis [25] The analyses were performed on fresh yoghurts to evaluate the samples at the stage of fully developed gel structure and stabilized post-fermentation acidification, while minimizing storage-related changes such as post-acidification and progressive structural rearrangements.

### 2.2. Yoghurt Analysis

#### 2.2.1. Acidity and Basic Composition

The pH of the yoghurts was measured before, during and after fermentation by pH-meter CP-401 (Elmetron, Zabrze, Poland). Potential acidity (°SH) was determined by the titration method and expressed as the content of lactic acid [27]. The yoghurts were analysed for content of protein (Kjeldahl method according to PN-EN ISO 8968-1:2004 [28]), fat (van Gulik’s method) and dry matter (oven-drying at 102 °C) [27]. The measurements were made in triplicate.

#### 2.2.2. Determination of Whey Proteins

In order to evaluate the content of the certain undenatured whey proteins, i.e., α-lactalbumin (α-LA), β-lactoglobulin (β-LG), bovine serum albumin (BSA), lactoferrin and lysozyme, 10 g of yoghurts were centrifuged at 1250× *g* for 10 min at 5 °C in a refrigerated high-speed centrifuge (Universal 320, Hettich, Kirchlengern, Germany). Obtained whey solutions were filtered through the paper quality filter discs—diameter: 125 mm, density: 65 g/m^2^, grade: 3 h (Munktell, Bärenstein, Germany) and then 0.20-μm disposable sterile filters (Millipore type GSTF, Burlington, MA, USA). The supernatants in vials were kept chilled in the fridge until further analysis and in the suitable time injected into the chromatograph (in the amount of 20 μL). The chromatographic analyses were performed using reversed-phase high-performance liquid chromatography (RP-HPLC) method [25]. The chromatographic conditions included a C18 column of 250 mm length and 4.6 mm diameter maintained at controlled temperature, UV detection, and gradient elution using acetonitrile and water containing trifluoroacetic acid as mobile phases. The flow rate was set at 1 mL/min. The total analysis time for a single sample was 35 min at 205 nm wavelength with column temperature of 37 °C. Calibration curves were prepared using analytical standards of purified proteins, i.e., α-LA (≥85%), β-LG (90%), BSA (≥96%) and lactoferrin (90%) all from bovine milk, as well lysozyme (95%) from hen egg whites, were purchased from Sigma-Aldrich (St. Louis, MO, USA). The analyses of reference substances were conducted under the same conditions. Quality control included duplicate injections, external calibration, and verification of peak retention times using reference standards. On the grounds of the obtained chromatograms, using program Star 6.2 Chromatography Workstation (Varian, Palo Alto, CA, USA), the qualitative and quantitative identification of each substance was performed followed by their concentration determination.

#### 2.2.3. Determination of Lipophilic Vitamins

RP-HPLC method was also used to determine the concentrations of lipophilic vitamins, i.e., A, D_3_ and E, and β-carotene. All samples were prepared by the Röse-Gottlieb fat extraction method, and 5 mL of yoghurts were used. Vitamin separation was performed by a liquid chromatograph ProStar 210 model, a UV-VIS ProStar 325 and fluorescence ProStar 363 detectors (Varian, Palo Alto, CA, USA). Mobile phase was a mixture of acetonitrile, dichloromethane, methanol and water for HPLC (Sigma-Aldrich, St. Louis, MO, USA). Measurements were carried out using the column PursuitXRs 3-C18 (Varian, Palo Alto, CA, USA) of 150 mm length and 4.6 mm diameter. Total analysis time for a single sample was 25 min, at the flow rate—1 mL/min. Quantitative determination of vitamins was performed using external standard calibration. The analyses of reference substances, i.e., (±)-α-tocopherol (≥97% HPLC) for vitamin E, cholecalciferol (≥98% HPLC)—vitamin D_3_, retinol (≥99% HPLC)—vitamin A and β-carotene (≥95% HPLC) (Sigma-Aldrich, St. Louis, MO, USA), were conducted under the same conditions. Vitamin A was determined using fluorescence detection at an excitation wavelength of 330 nm and an emission wavelength of 470 nm. Vitamin E was analysed using fluorescence detection at excitation and emission wavelengths of 295 nm and 330 nm, respectively. Vitamin D_3_ was determined using UV detection at 265 nm, whereas β-carotene was simultaneously analysed using UV-Vis detection at 450 nm. On the grounds of the obtained chromatograms, the qualitative and quantitative identification of each substance was performed [25]. Detector wavelengths were adjusted according to the specific absorption characteristics of the analysed compounds. The analytical procedure included quality-control verification based on standard solutions, duplicate measurements, and assessment of chromatographic repeatability.

#### 2.2.4. Texture

Texture parameters of the yoghurt curds and the viscosity of the yoghurts were measured using a BDO-FB0.5TS universal testing machine (Zwick GmbH and Co., Ulm, Germany). Texture parameters (firmness/hardness, consistency/adhesiveness, cohesiveness, springiness, gumminess, chewiness and cohesive strength) were tested after about 20 h of storage at 4–6 °C. The yoghurt curds were placed in a beaker with an inner diameter of 50 mm and compressed twice with a cylindrical die (45 mm diameter, 5 mm height) to a depth of 25 mm and at a speed of 1 mm/s. The second compression cycle was preceded by a 2-s relaxation phase. Apparent viscosity was determined using a dedicated back-extrusion set (beaker with 50 mm diameter and 60 mm height, plunger with 45 mm diameter). The viscosity parameter (Pa·s) was calculated based on the force required for sample flow through the annular space between the plunger and the beaker during the test. Mean values were calculated from 2 cycles (at crosshead speeds of 50 and 100 mm/min). The measurements were made in duplicate. All measurements were carried out in duplicate, and the obtained data were processed using testXpert II software v 3.61 (Zwick GmbH and Co., Ulm, Germany).

#### 2.2.5. Instrumentally Measured Colour

The colour of yoghurt samples was measured by a Minolta CR-310 Chroma Meter (Minolta Camera Co. Ltd., Osaka, Japan) using D65 as the standard light source. Yoghurt samples were poured into small disposable petri dishes (60 mm in diameter, height layer of the sample was 10 mm), and then put onto a white standard plate. The reflectance of yoghurt surface was measured using the measuring head (50 mm diameter of aperture; geometry 0°). The CIE colour parameters were L* (lightness), a* (redness/greenness) and b* (yellowness/blueness) [25]. Moreover, the additional colour parameters were calculated based on the measured CIE L*a*b* coordinates. Chroma (C*) was determined as a measure of colour saturation using the Equation (1):

C* = √((a*^2^ + b*^2^)). (1)

The hue angle (h°), describing the colour tone, was calculated according to the Equation (2) as:

h° = atan2 (b*, a*),(2)

because negative a* values were observed, and expressed in degrees. The whiteness index (WI), reflecting the perceived whiteness of the yoghurt, was calculated according to the Equation (3):

WI = 100 − √((100 − L*)^2^ + a*^2^ + b*^2^)). (3)

Measurements were performed at refrigerated temperature (approx. 6 °C), using D65 illuminant and standard observer angle of 2°. The instrument was calibrated against a white calibration plate before analysis. Four readings were performed for each sample.

### 2.3. Statistical Analysis

Statistical analysis was performed using StatSoft Inc. Statistica ver. 13.3 software (Dell Inc., Round Rock, TX, USA) [29]. The results are presented as mean ± standard deviation (SD), together with additional descriptive statistics, including minimum and maximum values, coefficient of variation (CV), and range, in order to assess data dispersion and variability of the analysed parameters. The experimental unit was the independent yoghurt batch, and analytical replicates were averaged prior to statistical analysis. The assumptions of normality and homogeneity of variance were verified using the Shapiro-Wilk and Levene tests, respectively. Relationships between variables were evaluated using Pearson’s linear correlation coefficients (r). Correlation analyses were performed for: (i) basic physicochemical parameters and texture and colour attributes; (ii) bioactive components of protein and fat fractions; and (iii) relationships between bioactive components, and analysed texture and colour parameters. The strength of correlations was interpreted based on the absolute value of Pearson’s coefficient (|r|), irrespective of the sign of the relationship, as follows: weak (|r| < 0.3), moderate (0.3 ≤ |r| < 0.7), and strong (|r| ≥ 0.7). Results at *p* ≤ 0.05 were considered statistically significant.

Multiple linear regression analysis was additionally performed to identify physicochemical variables associated with selected texture parameters (firmness and apparent viscosity) and colour attributes (lightness and whiteness index). Regression analyses were conducted using selected physicochemical variables as independent predictors. The results are presented as regression coefficients (β) together with standard errors (SE). The explanatory power of the regression analyses was evaluated using the adjusted coefficient of determination (adjusted R^2^). Statistical significance of predictors was verified at *p* ≤ 0.05. Regression assumptions were assessed based on residual analysis.

The coefficient of variation (CV) was used as an additional measure of relative variability, supporting interpretation of dataset heterogeneity and reliability, particularly for texture parameters characterized by greater dispersion. Due to the relatively large number of calculated correlations, the obtained *p*-values were interpreted cautiously and additionally evaluated in the context of biological and technological relevance of the observed relationships.

## 3. Results and Discussion

### 3.1. Physicochemical Characteristics

Physicochemical characteristics as well texture and colour parameters of organic natural yoghurts included into the study were presented in Table 1 and Table 2.

The analysed yoghurts showed pH and lactic acid values typical of properly fermented yoghurt, with mean values of 4.57 and 0.98%, respectively. The composition of the samples was relatively stable, as indicated by low coefficients of variation for pH, total protein, fat, and most bioactive components. In particular, pH exhibited very low variability (CV = 2.41%), showing a relatively uniform fermentation process, whereas lactic acid (CV = 11.22%) and potential acidity (CV = 8.54%) showed greater variability, which may reflect differences in fermentation kinetics. Protein and fat fractions remained relatively stable (CV mostly below 8%), while lipophilic vitamins and β-carotene exhibited greater variability, likely related to their susceptibility to oxidation and processing-related losses. Texture parameters showed greater variability than compositional traits. Firmness (CV = 10.98%) and apparent viscosity (CV = 8.79%) exhibited moderate variability, whereas secondary texture parameters such as springiness (CV = 14.04%), chewiness (CV = 15.38%), and cohesiveness (CV = 19.44%) were more variable, possibly reflecting differences in gel microstructure and protein-fat interactions occurring in yoghurt production. As derived parameters, their variability represents cumulative structural effects and should, therefore, be interpreted with caution. Colour parameters were comparatively stable, particularly L* and WI, whereas b* showed wider variation, consistent with differences in fat-associated pigments and lipophilic compounds. Lightness (L*) exhibited extremely low variability (CV = 0.51%), confirming high visual uniformity of the product. Range analysis supported these observations, with the largest differences observed for apparent viscosity, consistency, and b*, indicating technologically and sensorially relevant variability, whereas minimal variation in pH and L* suggested good analytical and technological repeatability. Overall, the descriptive statistics indicate that the dataset showed sufficient variability to evaluate associations among composition, texture, and colour parameters.

### 3.2. Relationship Between Parameters

#### 3.2.1. Basic Physicochemical Parameters and Texture Parameters

The correlation analysis showed that relationships between physicochemical parameters, and texture and colour attributes of organic plain yoghurts were selective and dependent on the specific parameter (Table 3).

Active acidity (pH) was positively correlated with firmness (r = 0.484, *p* ≤ 0.05) and moderately associated with apparent viscosity (r = 0.416)—Table 3, although the strength of this relationship remained limited due to the narrow pH variability (CV = 2.41%), as shown in Table 1. These findings are generally consistent with previous studies, indicating that acidification promotes casein aggregation and gel strengthening [30,31]. However, no significant relationships were observed between pH and most secondary texture parameters, suggesting that final acidity alone does not fully reflect gel structure development. This observation may be explained by the fact that yoghurt gel properties depend not only on acidification, but also on protein denaturation, protein-protein interactions, water-binding capacity, and the structural organisation of the gel matrix. As demonstrated by Xiong et al. [32] temperature-induced denaturation significantly modifies protein interactions, aggregation behaviour, and water-binding capacity, thereby influencing texture independently of pH value. Moreover, the relatively narrow pH range observed in this study (Table 1) further limits variability and reduces the strength of detectable correlations. Nevertheless, the obtained pH values remained within the typical range for yoghurt, close to the isoelectric point of casein (pI ≈ 4.6), where acid-induced gel formation occurs and post-fermentation acidification is typically minimized by rapid cooling, leading to stabilization of the yoghurt structure. This interpretation is consistent with the current understanding of yoghurt rheology. Le Ba et al. [26] reported that the decrease in pH during fermentation promotes casein aggregation and formation of a network of interconnected strands that entrap water, whey proteins, and fat globules, thereby contributing to yoghurt body, firmness, and viscosity. The Authors emphasized that yoghurt texture is additionally influenced by milk composition, total solids, starter culture, homogenization, and post-fermentation handling, which may explain why endpoint pH alone was insufficient to account for the variability of most secondary texture parameters in the present study. They also underlined that for semi-solid systems such as yoghurt, “firmness” is a more appropriate descriptor than “hardness”.

Total protein content showed a significant positive correlation not only with apparent viscosity (r = 0.480, *p* ≤ 0.05), but also with firmness (r = 0.510, *p* ≤ 0.05), indicating its contribution to both gel strength and resistance to flow. This result is in agreement with recent studies demonstrating that increased protein concentration enhances gel density and mechanical strength [30,33,34]. Moderate non-significant associations with cohesive strength (r = 0.458) and springiness (r = 0.395) were also observed. These relationships are consistent with literature showing that higher protein content may increase gel density and mechanical stability, although the final texture is also affected by processing and microstructural factors [26]. Protein concentration may significantly influence elasticity only under specific structural arrangements.

Fat content showed moderate but non-significant correlations with texture parameters, including consistency (r = 0.398) and apparent viscosity (r = 0.354), indicating a consistent trend despite lack of statistical significance. This is partially consistent with previous findings on the role of fat in enhancing creaminess and viscosity [35,36]. However, the lack of significant relationships contrasts with recent studies, which emphasize the importance of fat-protein interactions and fat globule size distribution in determining texture [37]. Thus, in the present dataset, fat content alone did not explain textural variability. Therefore, fat-related effects on texture may depend more on fat globule size, homogenization, and protein-fat interactions than on total fat concentration alone. Texture formation in yoghurt is primarily governed by protein interactions within the gel matrix, whereas fat contributes mainly through modulation of viscosity and sensory perception.

In contrast, colour parameters showed strong and consistent associations with fat content (Table 3). Significant negative correlations were observed for L* (r = −0.802, *p* ≤ 0.05) and WI (r = −0.820, *p* ≤ 0.05), whereas positive correlations were found for b* (r = 0.752, *p* ≤ 0.05) and C* (r = 0.741, *p* ≤ 0.05). These results indicate that higher fat content was associated with lower lightness (L*) and whiteness index (WI), but greater yellowness (b*) and chroma (C*). This pattern is consistent with the presence of lipophilic pigments and fat-associated compounds contributing to the optical properties of yoghurt. The obtained relationships confirm that colour variation in dairy products occurs mainly along the yellow-blue axis, as no significant correlations were observed for a* or h°. Similar findings were reported by Borba et al. [38], Chudy et al. [39], and Manzi et al. [40], who demonstrated that fat and lipophilic pigments, particularly β-carotene, are major contributors to yoghurt colour characteristics.

Generally, the correlations indicate that protein-related variables were more closely associated with selected texture parameters, whereas fat content showed stronger and more consistent associations with colour attributes.

#### 3.2.2. Whey Proteins and Lipophilic Vitamins

Investigating the relationships between individual bioactive milk constituents is important for linking specific whey proteins and lipophilic vitamins with the texture and colour characteristics of organic plain yoghurts, thereby improving understanding of their potential technological and functional roles. Such relationships may support the development of yoghurt products that are both nutritionally valuable and attractive to consumers. Therefore, Pearson’s linear correlation coefficients were calculated for bioactive components within the protein and fat fractions (Table 4) to evaluate the strength and direction of their associations. Significant positive correlations were found between β-lactoglobulin and vitamin D_3_ (r = 0.855, *p* ≤ 0.05) and between β-lactoglobulin and vitamin A (r = 0.809, *p* ≤ 0.05). These associations are consistent with the known ability of β-lactoglobulin to bind hydrophobic ligands, including retinol, vitamin D_3_, long-chain fatty acids, and cholesterol [41].

Significant (*p* ≤ 0.05) positive associations were also observed between lactoferrin and vitamin D_3_ (r = 0.721), vitamin A (r = 0.797), and β-carotene (r = 0.746), as well as between lysozyme and β-carotene (r = 0.634). These relationships may reflect co-variation of bioactive compounds within the milk matrix rather than direct causal interactions. The obtained results are in agreement with previous studies showing interactions between milk proteins and lipophilic vitamins. Positive associations between β-lactoglobulin and retinol [42], as well as between β-carotene and vitamins A and E [43], reflect the role of milk proteins as carriers of lipophilic compounds. In particular, whey proteins, including whey protein isolates (WPI), provide hydrophobic binding sites that facilitate ligand transport and stabilization. Recent studies have shown that food macromolecules, including proteins, lipids, and carbohydrates, enhance the stability and bioavailability of lipophilic vitamins through binding and encapsulation mechanisms [41].

Because the yoghurts were produced using heat treatment at 85 °C for 30 min, the measured whey proteins should be interpreted as residual native, undenatured or soluble fractions remaining after processing. Various reactions occur between milk constituents during thermal treatment, including whey protein denaturation, aggregation, and formation of protein complexes. Literature data indicate that such thermal conditions can induce extensive denaturation of heat-labile whey proteins, particularly lactoferrin, immunoglobulins, and β-lactoglobulin, and may alter their ligand-binding capacity and interactions with fat-soluble compounds [31,44,45,46,47,48]. β-Lactoglobulin is considered one of the most heat-sensitive whey proteins and readily undergoes thermal denaturation [31,46,47,48]. Previous studies demonstrated that increasing heat treatment intensity progressively reduces the content of residual native whey proteins, including β-lactoglobulin, α-lactalbumin, and lactoferrin [46,47,48]. Hammershøj et al. [49] reported limited β-lactoglobulin denaturation after HTST treatment (72 °C/15 s), whereas substantially higher denaturation occurred at elevated temperatures. Similarly, Sakkas et al. [50] observed a marked decrease in native β-lactoglobulin concentration with increasing heating intensity. Thermal processing may additionally modify the biological activity and ligand-binding capacity of whey proteins, particularly their ability to interact with hydrophobic compounds, including fat-soluble vitamins and carotenoids [45].

Therefore, considering the relatively intensive heat treatment applied during yoghurt manufacture (85 °C/30 min), the detected whey protein levels likely represent residual soluble or partially native protein fractions remaining after processing. Nevertheless, despite partial denaturation, these proteins may still contribute to protein-protein interactions, gel microstructure formation, water-binding capacity, emulsion stability, and light-scattering properties, thereby remaining associated with selected texture and colour parameters of yoghurt analysed in the present study. These relationships are additionally supported by studies demonstrating interactions between whey proteins and lipophilic compounds. Positive associations between β-lactoglobulin and retinol, as well as between β-carotene and vitamins A and E, reflect the ability of whey proteins to bind and stabilize hydrophobic ligands [41,42,43].

The observed associations are therefore consistent with previous reports on interactions between milk proteins and lipophilic compounds. However, due to the correlative nature of the study, these results should be interpreted as indications of compositional co-variation and potential matrix interactions rather than evidence of direct binding mechanisms in the analysed yoghurts.

#### 3.2.3. Whey Proteins and Lipophilic Vitamins, and Texture Parameters

Texture formation in yoghurt is primarily related to the organisation of the protein gel network, interactions between caseins and whey proteins, and the distribution of fat and water within the matrix. The texture of dairy products is closely linked to the interactions between milk proteins (casein and whey proteins) as well other components such as vitamins and exopolysaccharides. The quality of yoghurt gel is determined by the appropriate ratio of casein to whey proteins in milk, as well as by their molecular state [34,35,51]. This mechanism is further supported by Le Ba et al. [26] who showed that the rheological, microstructural, and textural properties of yoghurt are influenced by milk origin, total solids, starter culture, fat content, homogenization, whey protein addition, and post-fermentation handling. In particular, increases in total solids and protein enrichment were associated with enhanced firmness, complex viscosity, and gel strength, whereas post-fermentation stirring reduced apparent viscosity through disruption of the gel network. These findings support the interpretation that the correlations identified in the present study reflect not only compositional effects, but also the structural organization of the protein-fat matrix. These interactions not only influence the structural properties of dairy products but also their nutritional benefits and stability [34,35,51]. Understanding these relationships can help in optimizing the texture and functionality of dairy products, similarly to above discussed interactions among bioactive components.

In the present study, correlations between individual whey proteins, lipophilic vitamins, β-carotene, and texture parameters were evaluated to identify which components were most closely associated with instrumental texture attributes—Table 5.

Selected components of the protein fraction were associated with specific texture attributes. Springiness showed negative correlations with α-lactalbumin (r = −0.776, *p* ≤ 0.05) and BSA (r = −0.602, *p* ≤ 0.05), while β-lactoglobulin was positively associated with apparent viscosity (r = 0.705, *p* ≤ 0.05). Firmness was significantly associated only with α-lactalbumin (r = 0.577, *p* ≤ 0.05). Lactoferrin and lysozyme showed significant negative associations with cohesive strength (r = −0.625 for lactoferrin and r = −0.702 for lysozyme) and adhesiveness (r = −0.636 and r = −0.673, respectively), but positive associations with gumminess (r = 0.598 and r = 0.577, respectively).

The association between β-lactoglobulin and apparent viscosity is consistent with the role of whey proteins in strengthening fermented milk gels through heat-induced denaturation and interactions with casein micelles. However, the present results also show that secondary texture attributes, such as gumminess and cohesiveness, were not associated exclusively with β-lactoglobulin or α-lactalbumin, but had stronger relationships with lactoferrin, lysozyme, β-carotene, and selected lipophilic vitamins. Therefore, individual whey proteins may contribute differently to yoghurt gel structure and texture development rather than exerting a uniform effect on gel properties. The obtained relationships are consistent with previous studies demonstrating the technological importance of whey proteins in fermented dairy products. Whey proteins are widely used in yoghurts and yoghurt-like products to improve texture, increase total solids, and enhance firmness [34,52]. Lesme et al. [34] reported that the addition of whey protein aggregates significantly modified yoghurt microstructure, resulting in increased gel strength, viscosity, and water-holding capacity. These structural changes were closely associated with instrumental texture parameters, indicating that protein-protein interactions within the gel matrix may influence springiness, cohesiveness, and creaminess of fermented dairy products. Similarly, Bierzuńska et al. [52] demonstrated that incorporation of polymerized whey proteins into probiotic yoghurt improved textural stability during refrigerated storage by increasing water-holding capacity, cohesiveness, and viscosity index, while reducing whey syneresis. According to these Authors, targeted modification of protein interactions within the yoghurt matrix may represent an effective approach for modulating rheological and sensory properties of fermented dairy products.

Yoghurt textural characteristics are influenced not only by proteins but also fat content is a critical factor influencing its texture, viscosity [35,36]. In food products, fat acts as structuring materials, and it is also an excellent solvent for flavour compounds, which are mostly hydrophobic. Although total fat content showed only moderate non-significant relationships with texture, β-carotene and selected fat-soluble vitamins were positively associated with gumminess, chewiness, cohesiveness, and apparent viscosity. These associations may be linked to the distribution of lipophilic compounds in the fat phase and to their indirect relationship with the microstructure of the yoghurt matrix. Kaminarides et al. [36] reported higher sensory acceptance of high-fat sheep milk yoghurts (6.6% and 3.8% versus 2.3% or 0.9%), suggesting that the effect of fat on texture and flavour is strongly dependent on milk type and processing conditions, particularly homogenization. According to Nami et al. [53], the relationship between vitamin content and yoghurt texture is influenced by the type and concentration of vitamins added. While some vitamins, such as those used in heart-healthy formulations, may not significantly alter texture, others like encapsulated vitamin D_3_ can enhance viscosity and reduce syneresis. Additionally, the use of hydrocolloids and other nutrient-rich additives can significantly improve the textural properties of yoghurt, leading to better consumer acceptance. However, since no sensory evaluation was performed, references to consumer acceptance should be treated only as literature-based context.

In the present study, it was therefore considered justified to calculate correlations between components of the fat fraction—namely lipophilic vitamins and textural parameters, as such relationships have been very rarely reported in the literature. Among the fat-fraction components, β-carotene showed the strongest associations with selected texture parameters, particularly gumminess (r = 0.776, *p* ≤ 0.05), chewiness (r = 0.733, *p* ≤ 0.05), and cohesiveness (r = 0.710, *p* ≤ 0.05), while it was negatively associated with cohesive strength (r = −0.760, *p* ≤ 0.05). Vitamin A (r = 0.666), vitamin D_3_ (r = 0.715), and vitamin E (r = 0.582) were associated mainly with apparent viscosity, and vitamins A and D_3_ were also correlated with gumminess (r = 0.655 and r = 0.607, respectively). These findings indicate that the texture of organic yoghurt was related not only to whey proteins, but also to selected components of the fat fraction. Nevertheless, these correlations should not be interpreted as causal effects.

#### 3.2.4. Whey Proteins and Lipophilic Vitamins, and Colour Parameters

Protein and fat fractions, including their bioactive components, were also associated with yoghurt colour parameters [39,40,54,55,56,57,58]. In the present study, fat content was negatively correlated with L* (r = −0.802, *p* ≤ 0.05) and WI (r = −0.820, *p* ≤ 0.05), and positively correlated with b* (r = 0.752, *p* ≤ 0.05) and C* (r = 0.741, *p* ≤ 0.05)—Table 3. Lightness was also negatively associated with several individual protein (β-lactoglobulin and lactoferrin) and fat-fraction (β-carotene, and vitamins A and D_3_) components, whereas b* and C* were positively related mainly to β-lactoglobulin, α-lactalbumin, vitamin A, and vitamin D_3_ (Table 6).

The obtained results indicate that colour parameters were associated with both fat and protein fractions, although the patterns differed between these groups. In the case of the fat fraction, lipophilic compounds, particularly vitamin A, vitamin D_3_, and β-carotene, were negatively associated with lightness (L*) and whiteness index (WI), while showing positive relationships with b* and chroma (C*). These relationships suggest that higher concentrations of lipophilic compounds were related to lower lightness and whiteness, but greater yellowness and colour saturation, which is consistent with their absorption properties and distribution within the fat phase. In contrast, relationships involving β-lactoglobulin and lactoferrin were mainly associated with negative correlations with L* and WI, and positive correlations with b*. These effects may be linked less to direct pigment contribution and more to indirect modifications of yoghurt microstructure and light-scattering properties within the protein-fat matrix.

In the present study, higher fat content was associated with lower lightness and whiteness, but higher yellowness. This is consistent with the role of fat-associated pigments and lipophilic compounds in modifying the optical properties of dairy matrices. Literature data indicate that carotenoids and fat-soluble vitamins can increase yellowness, while proteins may affect light scattering through their contribution to the gel network and colloidal structure. Fat content strongly influences the colour and overall visual quality of yoghurt [58]. Lightness tends to decrease with increasing concentrations of pigmented compounds, whereas yellowness increases due to their preferential partitioning into the fat phase [40]. Proteins, including β-lactoglobulin, α-lactalbumin, and lactoferrin, may additionally modulate colour parameters by influencing light scattering and stabilizing pigment distribution within the milk matrix.

Manzi et al. [40] reported a strong positive correlation between β-carotene content and b* values in whole milk (r = 0.854), indicating that higher carotenoid concentrations increase product yellowness. Similar relationships were reported by Madora et al. [56] in studies on yoghurt fortified with carotenoid-rich ingredients. The addition of carrot powder, a source of β-carotene and provitamin A, resulted in a significant decrease in lightness (L*) and an increase in redness (a*) and overall colour intensity with increasing enrichment levels. The highest addition level (3%) produced the lowest L* values and the greatest colour changes, confirming that incorporation of carotenoid-rich components directly modifies the optical properties and colour saturation of yoghurt.

The importance of fat-soluble compounds for colour formation is also supported by the review of Chudy et al. [39], who emphasized that colour measurement in the CIE Lab* system enables objective assessment of lightness, redness-greenness, and yellowness-blueness in dairy products. According to these Authors, colour parameters may be influenced by fat content, carotenoids, vitamins, and protein composition through both pigment-related effects and light scattering within the colloidal structure of milk. Therefore, instrumental colour measurement may be useful for assessing product quality and monitoring changes occurring during storage [39,54,56,59,60]. However, since sensory analysis was not performed in the present study, statements concerning consumer preference should be interpreted only in the context of previously published literature.

Generally, both the present study and previous reports indicate that strong correlations between L*, b*, WI, and selected fat-soluble compounds are associated primarily with the fat fraction rather than the protein fraction. These relationships likely reflect both pigment-related optical effects and matrix-related light scattering within the yoghurt structure.

#### 3.2.5. Correlation Structure and Heatmap Visualization of Relationships Among Parameters

**Figure 1:**
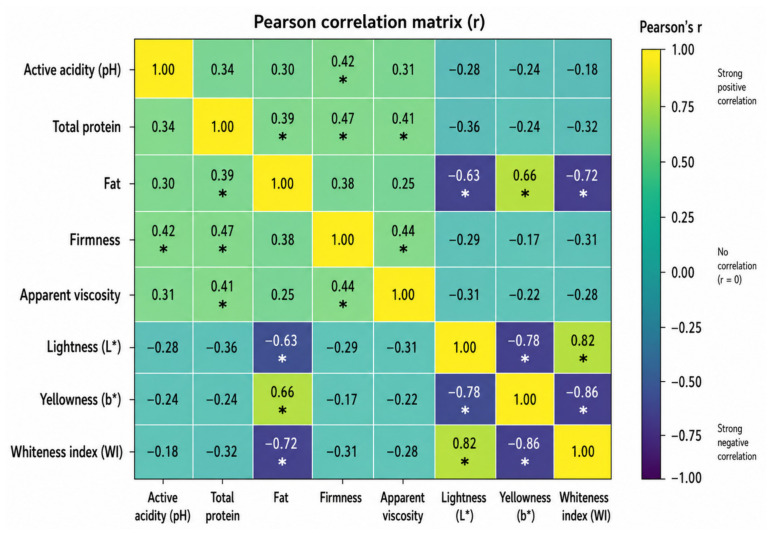
Heatmap of Pearson’s correlation coefficients (r) between selected physicochemical parameters, texture attributes, and colour characteristics of organic plain yoghurts. Colour scale ranges from −1 to +1. Positive correlations are shown in yellow-green colours, whereas negative correlations are represented by blue-purple colours. Colour intensity reflects the strength of the correlation coefficient. *—statistically significant correlations at *p* ≤ 0.05.

**Figure 2:**
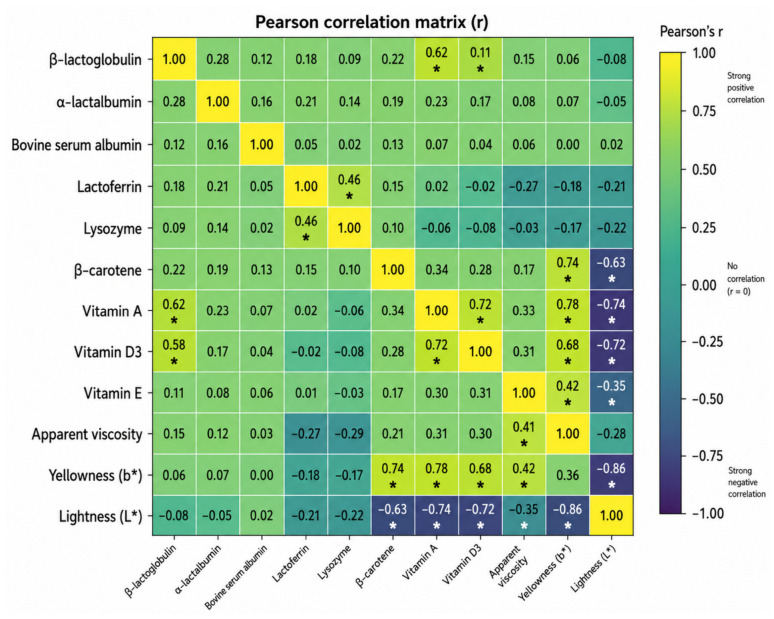
Heatmap of Pearson’s correlation coefficients (r) between whey proteins, lipophilic vitamins, texture attributes, and colour characteristics of organic plain yoghurts. Colour scale ranges from −1 to +1. Positive correlations are shown in yellow-green colours, whereas negative correlations are represented by blue-purple colours. Colour intensity reflects the strength of the correlation coefficient. *—statistically significant correlations at *p* ≤ 0.05.

A comprehensive overview of associations among the analysed parameters is presented in Figure 1 and Figure 2 as correlation heatmaps. Heatmap-based visualization enabled identification of dominant association patterns and clustering of variables that were not immediately evident from pairwise correlations alone. Figure 1 illustrates the overall correlation pattern between physicochemical composition, texture, and colour parameters. Two partially distinct relationship clusters were observed, corresponding mainly to texture-associated variables and colour-associated variables. Texture-related variables clustered mainly with total protein, whereas colour parameters (L*, b*, and WI) formed a separate module associated with fat content. The position of active acidity—pH outside these two main clusters suggests that endpoint acidity had a more limited contribution to the overall correlation structure than protein or fat contents.

Whey proteins and lipophilic vitamins formed partially overlapping clusters (Figure 2), suggesting compositional co-variation and potential matrix interactions. β-lactoglobulin was closely associated with vitamins A and D_3_, which is consistent with its potential role as a carrier of hydrophobic ligands. Lactoferrin and lysozyme showed a distinct pattern linked to selected texture parameters which appears to be of limited functional relevance for yoghurt texture formation, whereas β-carotene and lipophilic vitamins were more closely associated with colour attributes, particularly L* and b*.

The separation between the protein-texture and fat-colour modules suggests structural modularity of the yoghurt matrix. Texture parameters were more closely related to protein network organisation and gel structure, whereas colour attributes were more strongly associated with the optical properties of the fat phase and associated lipophilic compounds. This modular pattern supports the interpretation that yoghurt texture and colour are linked to partly independent compositional and structural features.

### 3.3. Multiple Regression Analysis

Multiple regression analysis was performed to evaluate the extent to which selected physicochemical variables were associated with firmness, apparent viscosity, lightness (L*), and whiteness index (WI) of the analysed yoghurts—Table 7.

The regression analyses for firmness and apparent viscosity showed moderate explanatory power (adjusted R^2^ = 0.58 and 0.63, respectively), indicating that compositional variables explained only part of the observed textural variability. Firmness was primarily associated with total protein concentration and active acidity (to a lesser extent), supporting the role of protein content and acid-induced casein aggregation in yoghurt gel formation. Apparent viscosity was mainly related to total protein content and β-lactoglobulin concentration, suggesting that whey protein interactions and increased total solids contribute to flow resistance and gel stabilization. Nevertheless, a substantial proportion of texture variability remained unexplained, suggesting that additional factors, including yoghurt microstructure, starter culture activity, protein denaturation, fat globule size distribution, homogenization efficiency, and post-fermentation structural rearrangements, may also contribute to texture development.

Colour-related parameters showed, however, stronger predictive performance. The regression analysis for lightness (L*) revealed relatively high explanatory power (adjusted R^2^ = 0.71). Fat and vitamin A contents were negatively associated with L*, indicating that increasing concentrations of lipophilic compounds were related to reduced lightness and enhanced colour saturation. The analysis for whiteness index (WI) showed high explanatory power (adjusted R^2^ = 0.69), with significant negative associations observed for fat and β-carotene. These findings indicate the contribution of fat-associated pigments and lipophilic compounds to the optical properties of yoghurt. Generally, the regression analysis suggests that colour parameters were more strongly associated with fat-soluble compounds, whereas texture parameters appeared to depend on a broader range of compositional and structural factors.

Regression coefficients are presented together with standard errors (SE). All predictors included in the analysis were statistically significant at *p* ≤ 0.05. Regression assumptions were verified by residual analysis. Because the study was observational and correlation-based, the regression analysis should be interpreted as exploratory predictive associations rather than evidence of direct biological relationships.

## 4. Conclusions

The study showed that selected bioactive components of the protein and fat fractions were associated with instrumental texture and colour parameters of organic plain yoghurts. Whey proteins, particularly β-lactoglobulin and α-lactalbumin, were related mainly to apparent viscosity, firmness, and springiness, whereas gumminess showed stronger associations with lactoferrin, lysozyme, β-carotene, vitamin A, and vitamin D_3_. Colour parameters were more closely related to fat-fraction components, especially vitamin A, vitamin D_3_, β-carotene, and total fat, which were associated with lower lightness and whiteness, and higher yellowness and chroma. Regression analysis indicated that firmness and apparent viscosity were only moderately explained by the selected compositional predictors, whereas WI was more strongly associated with fat and vitamin A content. The remaining variability is likely related to microstructural and processing-related factors, including heat treatment, homogenization, fermentation dynamics, and post-fermentation gel rearrangement. Due to the correlative design of the study, the results should be interpreted as associations rather than direct causal effects. Nevertheless, they provide a basis for further research on the functional role of whey proteins and fat-soluble vitamins in shaping instrumental quality traits of organic yoghurt. Generally, the findings indicate that texture is predominantly protein-driven, colour is fat-driven, and the remaining variability can be attributed to microstructural factors within the yoghurt matrix, highlighting the potential to optimize yoghurt formulations through targeted modification of composition and processing conditions.

## Figures and Tables

**Table 1:** Physicochemical characteristics of organic plain yoghurts.

Parameter	Mean ± SD	Minimum Value	Maximum Value	CV (%)	Range
Acidity					
Active acidity—pH value	4.57 ± 0.11	4.42	4.69	2.41	0.27
Potential acidity (°SH)	40.05 ± 3.42	34.63	49.74	8.54	15.11
Lactic acid (%)	0.98 ± 0.11	0.80	1.12	11.22	0.32
Protein fraction					
Total protein (%)	3.84 ± 0.17	3.31	4.04	4.43	0.73
β-lactoglobulin (g/L)	1.57 ± 0.13	1.32	1.87	8.28	0.55
α-lactalbumin (g/L)	0.90 ± 0.06	0.78	1.04	6.67	0.26
Bovine serum albumin (g/L)	0.42 ± 0.01	0.39	0.45	2.38	0.06
Lactoferrin (mg/L)	31.84 ± 2.71	26.53	37.15	8.51	10.62
Lysozyme (µg/L)	4.54 ± 0.23	4.09	5.03	5.07	0.94
Fat fraction					
Fat (%)	3.74 ± 0.21	3.38	4.06	5.61	0.68
β-carotene (mg/L)	0.295 ± 0.022	0.252	0.338	7.46	0.09
Vitamin A (mg/L)	0.434 ± 0.028	0.391	0.479	6.45	0.09
Vitamin D_3_ (µg/L)	0.754 ± 0.063	0.652	0.856	8.36	0.20
Vitamin E (mg/L)	1.712 ± 0.119	1.489	1.943	6.95	0.45

CV—coefficient of variation; Range—difference between maximum and minimum values.

**Table 2:** Texture and colour in the CIE L*a*b* colour space parameters of organic plain yoghurts.

Parameter	Mean ± SD	Minimum Value	Maximum Value	CV (%)	Range
Texture parameters					
Firmness/Hardness (N)	2.64 ± 0.29	2.27	3.19	10.98	0.92
Cohesive strength (N)	1.16 ± 0.12	0.86	1.32	10.34	0.46
Consistency/Adhesiveness (mJ)	8.82 ± 0.93	7.43	10.50	10.54	3.07
Springiness (–)	0.57 ± 0.08	0.41	0.72	14.04	0.31
Gumminess (N)	0.88 ± 0.10	0.67	1.14	11.36	0.47
Chewiness (N)	0.52 ± 0.08	0.41	0.68	15.38	0.27
Cohesiveness (–)	0.36 ± 0.07	0.28	0.44	19.44	0.16
Apparent viscosity (Pa·s)	13.09 ± 1.15	10.99	15.30	8.79	4.31
Colour parameters in the CIE L*a*b* colour space					
L*	89.79 ± 0.46	89.28	90.86	0.51	1.58
a*	−3.28 ± 0.15	−3.52	−3.02	4.57	0.50
b*	14.54 ± 1.36	11.14	16.87	9.35	5.73
C*	14.92 ± 1.25	12.62	17.19	8.38	4.57
h°	102.70 ± 3.86	95.87	109.38	3.75	13.51
WI	81.94 ± 1.10	79.80	83.91	1.34	4.11

CV—coefficient of variation; Range—difference between maximum and minimum values; CIE L*a*b* coordinates: L*—lightness, a*—redness/greenness, b*—yellowness/blueness, C*—chroma, h°—hue angle, WI—whiteness index.

**Table 3:** Correlation coefficients (r) between basic physicochemical parameters, and texture and colour parameters in the CIE L*a*b* colour space in organic plain yoghurts.

Parameter	Active Acidity—pH Value	Total Protein	Fat
Texture parameters			
Firmness/Hardness	0.484 *	0.510 *	0.223
Cohesive strength	0.393	0.458	−0.293
Consistency/Adhesiveness	0.271	0.221	0.398
Springiness	−0.172	0.395	−0.248
Gumminess	0.045	0.443	0.002
Chewiness	0.083	0.181	−0.117
Cohesiveness	0.147	0.199	−0.106
Apparent viscosity	0.416	0.480 *	0.354
Colour parameters in the CIE L*a*b* colour space			
L*	0.102	−0.376	−0.802 *
a*	−0.051	−0.109	−0.219
b*	−0.019	0.388	0.752 *
C*	0.095	0.360	0.741 *
h°	−0.120	−0.200	−0.410
WI	0.110	−0.390	−0.820 *

CIE L*a*b* coordinates: L*—lightness, a*—redness/greenness, b*—yellowness/blueness, C*—chroma, h°—hue angle, WI—whiteness index. *—significant at *p* ≤ 0.05.

**Table 4:** Correlation coefficients (r) between bioactive components of protein and fat fractions in organic plain yoghurts.

Specification		Protein Fraction				
		β-Lactoglobulin	α-Lactalbumin	Bovine Serum Albumin	Lactoferrin	Lysozyme
Fat fraction	β-carotene	0.397	0.272	0.261	0.746 *	0.634 *
	Vitamin A	0.809 *	0.519	0.454	0.797 *	0.460
	Vitamin D_3_	0.855 *	0.545	0.479	0.721 *	0.348
	Vitamin E	0.562	0.324	0.258	−0.049	−0.166

*—significant at *p* ≤ 0.05.

**Table 5:** Correlation coefficients (r) between components of protein and fat fractions, and texture parameters.

Texture Parameters	Protein Fraction Whey Proteins					Fat Fraction			
						β-Carotene	Lipophilic Vitamins		
	β-Lactoglobulin	α-Lactalbumin	Bovine Serum Albumin	Lactoferrin	Lysozyme		Vitamin A	Vitamin D_3_	Vitamin E
Firmness	0.256	0.577 *	0.385	−0.202	−0.512	−0.379	0.003	0.072	0.360
Cohesive strength	−0.190	0.234	0.038	−0.625 *	−0.702 *	−0.760 *	−0.490	−0.431	0.206
Adhesiveness	−0.071	0.084	−0.167	−0.636 *	−0.673 *	−0.497	−0.245	−0.178	0.237
Springiness	−0.508	−0.776 *	−0.602 *	−0.010	0.382	0.282	−0.258	−0.326	−0.420
Gumminess	0.371	−0.062	−0.070	0.598 *	0.577 *	0.776 *	0.655 *	0.607 *	−0.024
Chewiness	0.075	−0.376	−0.304	0.470	0.638 *	0.733 *	0.404	0.334	−0.198
Cohesiveness	0.118	−0.324	−0.215	0.526	0.676 *	0.710 *	0.439	0.372	−0.182
Apparent viscosity	0.705 *	0.578 *	0.524	0.541	0.213	0.305	0.666 *	0.715 *	0.582 *

*—significant at *p* ≤ 0.05.

**Table 6:** Correlation coefficients (r) between components of protein and fat fractions, and colour parameters in the CIE L*a*b* colour space.

Colour Parameters	Protein Fraction Whey Proteins					Fat Fraction			
						β-Carotene	Lipophilic Vitamins		
	β-Lactoglobulin	α-Lactalbumin	Bovine Serum Albumin	Lactoferrin	Lysozyme		A	D_3_	E
L*	−0.728 *	−0.410	−0.339	−0.717 *	−0.389	−0.757 *	−0.958 *	−0.741 *	−0.097
a*	−0.377	0.164	0.260	−0.064	−0.037	−0.475	−0.420	−0.489	−0.434
b*	0.854 *	0.645 *	0.569	0.557	0.130	0.582	0.901 *	0.954 *	0.449
C*	0.836 *	0.620 *	0.561	0.537	0.120	0.559	0.887 *	0.939 *	0.425
h°	−0.409	−0.175	−0.113	−0.211	−0.081	−0.427	−0.365	−0.427	−0.298
WI	−0.781 *	−0.433	−0.376	−0.748 *	−0.403	−0.810 *	−0.975 *	−0.946 *	−0.131

CIE L*a*b* coordinates: L*—lightness, a*—redness/greenness, b*—yellowness/blueness, C*—chroma, h°—hue angle, WI—whiteness index. *—significant at *p* ≤ 0.05.

**Table 7:** Multiple regression analysis for selected texture and colour parameters of organic plain yoghurts.

Parameter (Dependent Variable)	Significant Predictors	β Coefficient ± SE	*p*-Value	Adjusted R^2^
Firmness	Total protein	0.47 ± 0.12	0.004	0.58
	pH	0.18 ± 0.07	0.031	
Apparent viscosity	Total protein	1.84 ± 0.51	0.002	0.63
	β-lactoglobulin	0.96 ± 0.33	0.018	
Lightness (L*)	Fat	−1.37 ± 0.28	<0.001	0.71
	Vitamin A	−0.92 ± 0.24	0.009	
Whiteness index (WI)	Fat	−1.54 ± 0.31	<0.001	0.69
	β-carotene	−1.08 ± 0.27	0.006	

R^2^—coefficient of determination.

## Data Availability

The data presented in this study are available on request from the corresponding author. The data are not publicly available due to privacy restrictions.
